# Port Sediments: Problem or Resource? A Review Concerning the Treatment and Decontamination of Port Sediments by Fungi and Bacteria

**DOI:** 10.3390/microorganisms9061279

**Published:** 2021-06-11

**Authors:** Grazia Cecchi, Laura Cutroneo, Simone Di Piazza, Giovanni Besio, Marco Capello, Mirca Zotti

**Affiliations:** 1 DISTAV, University of Genoa, 26 Corso Europa, I-16132 Genoa, Italy; grazia.cecchi@edu.unige.it (G.C.); laura.cutroneo@edu.unige.it (L.C.); simone.dipiazza@unige.it (S.D.P.); mirca.zotti@unige.it (M.Z.); 2 DICCA, University of Genoa, 1 Via Montallegro, I-16145 Genoa, Italy; giovanni.besio@unige.it

**Keywords:** bioremediation, microfungi, microorganisms, bottom marine sediments, metals, hydrocarbons, biosurfactants, Interreg IT-FR Maritime GEREMIA Project

## Abstract

Contamination of marine sediments by organic and/or inorganic compounds represents one of the most critical problems in marine environments. This issue affects not only biodiversity but also ecosystems, with negative impacts on sea water quality. The scientific community and the European Commission have recently discussed marine environment and ecosystem protection and restoration by sustainable green technologies among the main objectives of their scientific programmes. One of the primary goals of sustainable restoration and remediation of contaminated marine sediments is research regarding new biotechnologies employable in the decontamination of marine sediments, to consider sediments as a resource in many fields such as industry. In this context, microorganisms—in particular, fungi and bacteria—play a central and crucial role as the best tools of sustainable and green remediation processes. This review, carried out in the framework of the Interreg IT-FR Maritime GEREMIA Project, collects and shows the bioremediation and mycoremediation studies carried out on marine sediments contaminated with ecotoxic metals and organic pollutants. This work evidences the potentialities and limiting factors of these biotechnologies and outlines the possible future scenarios of the bioremediation of marine sediments, and also highlights the opportunities of an integrated approach that involves fungi and bacteria together.

## 1. Introduction

Marine sediments and, in particular, harbour/port sediments, currently represent the main source of inorganic and organic contaminants [1,2]. These contaminants are especially due to industrial and anthropogenic activities that impact the port system [3]. When the accumulation of sediments becomes excessive in port seabeds, dredging actions are necessary. Moving sediments favours contaminant mobilisation and also affects the quality of seawater in the port as well as the areas adjacent to the port itself [4,5]. Dredging technologies generally comprise the excavation, remotion, transport ex situ, and storage of hazardous marine sediment materials [3,6]. The main contaminants in harbour sediments are represented by heavy metals, hydrocarbons, and polycyclic aromatic hydrocarbons (PAHs), due to industrial activities and oil spills [3,7]. As mentioned above, dredging actions greatly impact the marine environment, and also affect the ecosystems and biodiversity of neighbouring marine areas [8]. Recently, researchers have investigated the best technology to remediate port sediments in situ, as a means to improve their quality to reuse them in industrial activities or nourishment [1,9,10].

As a result of this complex scenario, several research and application projects have been funded by the European Community to find efficient processes for the management, remediation, and reuse of marine sediments. Some of these projects have been set up under the European Interreg Programme, including: the SURICATES Project—Sediment Uses as Resources In Circular And Territorial EconomieS (Interreg North-West Europe)—that tests eco-innovative techniques in real-life conditions, providing long-term impact evaluation and guidelines for replication; the SETARMS Project—Sustainable Environmental Treatment and Reuse of Marine Sediment—that aims to find sustainable, economical, and environmental solutions for dredged sediment management; the VALSE Project—Interreg V France-Wallonie-Vlaanderen—that is intended to validate cross-border value-added sectors and participates in demonstrating the feasibility of reusing materials and the benefits of the circular economy; the PRISMA Project—Promoting Integrated Sediment Management (Interreg IV a 2 Mers Seas Zeeën)—that aims to develop improved methods for processing, treatment, and reuse of sediments in estuaries and coastal waterways from dredging to recycling; the SEDITERRA Project—Guidelines For the Sustainable Management of Dredged Sediments in the Maritime Area (Interreg Marittimo-IT FR-Maritime)—that aims to carry out pilot activities for the treatment of dredged sediments to allow their reuse and valorisation in civil engineering; and the GEREMIA Project—Wastewater Management for the Improvement of Port Waters (Interreg Marittimo-IT FR-Maritime)—that aims at developing integrated management tools and innovative methods (such as mycoremediation) in the treatment of port waters to improve their quality and, consequently, the quality of port sediments.

The activity carried out within the GEREMIA Project, in which we were directly involved, gave us the opportunity to explore the panorama of port sediment treatments and summarise what has been done in this field to date, with reference to innovative approaches. The approaches we identified to treat contaminated sediments can be separated into two main groups: the traditional approach and the approach based on the principles of bioremediation with fungi and bacteria as protagonists.

In our review, after a short overview of the main traditional strategies and biotechnologies of sediment remediation, we deeply explore the bioremediation and mycoremediation studies on marine sediments polluted by both ecotoxic metals and organic pollutants, and show the main microorganisms employed among fungi and bacteria. Moreover, we evidence the potentialities of these microorganisms in the production of biosurfactants and the recent possibility to exploit them in the remediation processes of marine sediments. Finally, we outline the possible future scenarios of the bioremediation of marine sediments, highlighting the potentialities of an integrated approach that involves fungi and bacteria together.

More than 140 papers were analysed. In addition, in the last decade, some review papers have been published. In particular, many of these works have dealt with the potentialities and advantages of bioremediation processes without specifying the application environment, while a few papers have dealt with the possible strategies of bioremediation in marine environments (coasts, seawater, sediments), but limited to one type of contaminant (i.e., oil spills, total hydrocarbons, PAHs, metals). On the contrary, in this review, we aimed to collect all the information about bioremediation technologies of organic and inorganic pollutants mediated by microorganisms (fungi and bacteria) in marine sediments, in situ or ex situ, and also to compare the methods and to highlight the potentialities of biosurfactant application and of integrated approaches of bioremediation. The literature search was performed using ScienceDirect, Scopus, and Google Scholar databases.

## 2. Marine Environment Remediation: Traditional Approaches and New Methods Based on the Principles of Bioremediation

Generally, after dredging, contaminated marine sediments are treated by the so-called ‘traditional approaches’, which involve chemical–physical methods. The main techniques employed are: the separation of the coarse fraction, which is commonly less contaminated, from the fine fraction, which is generally more contaminated [11]; soil washing, which exploits solvents and reagents to allow the extraction of hydrophobic contaminants from the sediments followed by transfer into the washing liquid [12,13,14,15,16]; and thermal desorption, which uses heat to increase the volatility of contaminants, efficiently removing them from the sediments [16,17]. Chemical extraction and/or thermostabilisation are the most commonly employed methods in the remediation of marine sediments contaminated with heavy metals and/or organic pollutants [18]. Although widely used, it is well known that traditional methods can negatively affect and alter marine biodiversity and their environmental impact is typically high [19]. Thanks to the studies carried out on the biogeochemical cycles and biogeochemical transformations in marine bottom sediments, researchers have evidenced the central role played by microorganisms in these processes. Microbial communities, in fact, can alter the microenvironment in which they live and are metabolically active; they can change pH values and redox potential by the secretion of enzymes, organic acids, and secondary metabolites, influencing and determining mineral dissolution, metal mobilisation, biomineral production, and non-stable metal species formation [20,21,22]. Starting from these studies, we found that the employment of microorganisms and their metabolism in bioremediation processes of marine sediments could represent a promising and green alternative to the traditional methods.

The term ‘bioremediation’ includes sensu lato all the remediation/restoration methods in which organisms are employed (e.g., plants, bacteria, fungi, algae, etc.). This technology is characterised by advantages and disadvantages, as are the traditional methods. In particular, bioremediation is a green technology: it exploits organisms in a sustainable and eco-friendly way to decontaminate the environment. It is less expensive than traditional methods and its rate of remediation can be very high, but the decontamination process can take a very long time. However, researchers have studied a promising branch of bioremediation that utilises the processes mediated by microorganisms such as fungi and bacteria, exploiting their metabolism to change metal bioavailability, mobilisation, and solubilisation, and to degrade organic pollutants. Two of the main approaches typically employed in marine sediments depend on the specific conditions created to stimulate microbial metabolism: bioaugmentation, which comprises inoculation of microbial strains in the sediments, and biostimulation, which stimulates the metabolic activities of the native microbial communities by the inoculation of specific nutrients [23,24,25].

Several biotechnologies for the remediation of many pollutants have been studied, but researchers have recently concluded that contamination with multiple pollutants (organic and inorganic), such as in port sediments, should be treated by an integrated approach that combines bioremediation techniques. Moreover, native microbial communities of polluted environments appear to be the best candidate for efficient and effective pollutant bioremoval, thanks to their gradual adaptation to the presence of contaminants over time [23,26,27]. In extremely contaminated environments, such as toxic marine sediments, microbes have evolved tolerance and adaptation mechanisms for their survival. The study and exploitation of these genotypic and phenotypic characteristics are crucial for bioremediation research [24,28,29,30]. Indeed, these microorganisms can be a reservoir of enzymes and metabolites that are potentially useful in bioremediation reactions [31,32,33,34,35]. In particular, fungi and bacteria have been extensively investigated and studied for their remediation potential of toxic organic and/or inorganic compounds and for their metabolic and biochemical pathways [32,33,34,35,36]. As reported by Villela et al. [37], many studies have focused on the potential of microorganisms to remediate efficiently marine environments. Those authors analysed all the patent documents of petroleum bioremediation by microbes and selected 500 patent documents: 368 by bacteria, 24 by fungi and yeast, 1 by archaea, 1 using a microalgal strain, and 32 by mixed consortia. The genera most represented in the patents are *Pseudomonas* (114 patents), *Bacillus* (75), and *Rhodococcus* (60).

Overall, these results show how fungi and bacteria can be used profitably in new bioremediation protocols of marine sediments with a very low environmental impact.

## 3. Marine Sediments Contaminated by Ecotoxic Metals: Approach Based on Bioremediation Mediated by Fungi and Bacteria

Metals cannot be directly degraded into harmless compounds; in marine sediments they are present in different states and species and only the defined ‘bioavailable fraction’ of metals contributes to the toxic rate of the sediments. However, the bioavailable fraction is not a stable parameter because metal speciation is continually influenced by chemical reactions between sediments and interstitial waters [30].

As mentioned in Section 2, it is well known that fungi and bacteria have developed resistance mechanisms to adapt to heavy metal contaminants and these microorganisms could be promising for bioremediation processes of marine sediments, providing green and sustainable techniques, and decreasing environmental impacts due to traditional methods [35,38]. As previously mentioned, microbes can change metal chemistry, mobility, stability, and bioavailability through metabolic products [39]. Many mechanisms of microbial resistance to heavy metals are known: precipitation of metals in stable states (e.g., phosphates, carbonates, and/or sulphides); metal volatilisation via methylation; physical and chemical exclusion of metals by components in cell walls/membranes and microbial metabolism; and intracellular accumulation due to low molecular weight, cysteine-rich proteins, and consequent sequestration in ad hoc cell compartments such as vacuoles [25,31,36,40]. These microbial strategies to resist the effects of toxic metals have led to two main bioremediation strategies: biomobilisation and bioimmobilisation [36]. The first, typically used against heavy metal-contaminated sediment, consists of metal biomobilisation in a liquid medium (e.g., inoculating microbes and biostimulating native microorganisms) and consequent dissolution of metals, separating solid and liquid fractions [36,41]. One of the most common approaches employed for biomobilisation is bioleaching, which exploits biological redox reactions, acid secretion, and pH changes to transform metals into soluble states [39,42,43]. Chemoautotrophic bacteria and fungi [39,44] are commonly employed in bioleaching. On the contrary, the second approach, bioimmobilisation, exploits microorganisms to inactivate metal toxicity through the microbial capability to biosorp, to bioaccumulate, to bioprecipitate, and to biotransform metals. Bioimmobilisation aims to reduce metal mobility, bioavailability, and toxicity without completely removing them from sediments. Biosorption involves the association of soluble heavy metals with the cell wall or the membrane surface of microorganisms through many mechanisms, such as complexation, chelation, reduction, and precipitation [33,45,46]. The bioaccumulation capability comprises the active transport and storage of the metal within microbial cells due to metabolism [36,39] and/or low molecular weight chelating agents/proteins. The latter can also be specific and selective as in the case of the active energy-dependent transport by siderophores that bind iron (Fe). Bioprecipitation exploits microbial metabolism, decreasing the metal species solubility (e.g., hydroxides, phosphates, carbonates, and sulphides) [39,47]. Bioleaching comprises the dissolution of metallic minerals, which release associated metals by microorganismal activity [36,42,48]. Finally, biotransformation can chemically modify heavy metals, altering their toxicity and bioavailability [36,39,49].

### Fungi and Bacteria Involved in Bioremediation Studies of Ecotoxic Metals in Marine Sediments

Chemolithoautotrophic bacteria belonging to the genus *Acidithiobacillus* are usually employed in bioremediation treatments of marine sediments in situ and ex situ. These bacteria acidify marine waters, fostering the chemical aggregation of sulphide minerals contained in marine sediment and consequently solubilising heavy metals [1,50,51,52,53]. Similarly, other methods aim to stimulate native Fe and sulphur (S) oxidising bacteria in situ and in deposits of dredged sediments e.g., [1,11,54]. Compared to the past, we are currently moving towards the exploitation of microbial consortia rather than single strains, underlining the importance of employing different microbes and different metabolic pathways in these processes. Beolchini et al. [1], for example, compared and studied the bioaugmentation effects of different bacterial strains in dredged sediments contaminated with heavy metals. The selected consortia belonging to acidophilic–chemoautotrophic and acidophilic–heterotrophic bacteria can oxidise Fe/S fractions and reduce Fe/manganese (Mn) fractions, respectively. The authors hypothesised that these strains could co-grow and positively interact with each other, maximising the effect and the efficacy of the bioremediation treatment proposed. They confirmed their hypothesis: bioremediation carried out by exploiting only one of the two categories of bacteria allowed extracting up to the 50% of metals. Furthermore, the contemporaneous employment of both the oxidising and the reducing bacteria increased the yields of the metal bioavailability and extraction up to 90%. This new bioremediation approach developed by Beolchini et al. [1] highlights the important role played by the synergism of consortia of microbial strains in the removal of heavy metals and the additional advantage that it is independent from the availability of S. More recently, Dell’Anno et al. [55] tested the heavy metal synergic immobilisation potential of five native bacteria (*Halomonas* sp. SZN1, *Alcanivorax* sp. SZN2, *Pseudoalteromonas* sp. SZN3, *Epibacterium* sp. SZN4, and *Virgibacillus* sp. SZN7) isolated from contaminated marine sediments, highlighting that these bacteria can change the arsenic (As), lead (Pb), and cadmium (Cd) mobility and bioavailability, reducing their toxicity.

**Figure 1:**
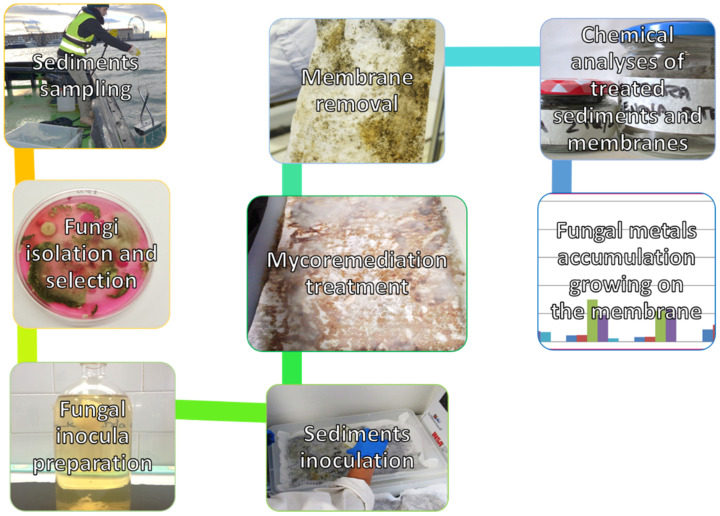
The sequence represents the main steps of the mycoremediation protocol developed and employed during the SEDITERRA Project.

Many fungi are also characterised by high heavy metal tolerance in marine environments. El-Kassas and El-Taher [56] isolated a metal-resistant *Trichoderma viride* Pers. strain from a heavy metal-polluted area in the Mediterranean Sea and proved that this fungus was able to biosorb and accumulate chromium (IV) (Cr(IV): 4.66 mg g^−1^ of chromium at pH 6 after 45 min). However, few studies have been carried out regarding the applicability of fungi in the mycoremediation of marine sediments. Thanks to the SEDITERRA Project (Section 1), Cecchi et al. [10,57] have developed and tested a new biotechnology to effectively exploit the bioaccumulation and biosorption of metals by microfungi from marine sediments. After isolating the autochthonous fungal strains from the sediments under study, they were used in the creation of specific co-inocula (consortia). The latter were grown on a tensile-resistant microporous membrane, which was subsequently made to adhere to the sediments to be decontaminated. The membrane must allow the fungi to grow easily in its texture, to absorb nutrients and metals from the underlying sediments, and to be removed easily at the end of the treatment, so that it can be disposed of as special waste, without leaving residues in the just-treated sediments. After 60 days of treatment, the membranes with fungi had hyperaccumulated numerous metals, including Cd, Cr, nickel (Ni), and copper (Cu) (Figure 1). Another microorganism known to remove metals from sediments is *Paradendryphiella salina* (G.K. Sutherland) Woudenb. And Crous, a marine fungus capable of biosorbing up to 80–92% of mercury (Hg) [58]. *Aspergillus* spp. (in particular, *Aspergillus candidus* Link, *Aspergillus flavus* Link, and *Aspergillus niger* Tiegh.) are very effective in mobilising metals and removing up to 90% of As from contaminated coastal waters [59,60]. Regarding the potential for metal mobilisation and removal by yeast, compared with filamentous fungi, they have been investigated only minimally. Some researchers have reported strains of *Yarrowia lipolytica* (Wickerham, Kurtzman and Herman) Van der Walt and Arx can remove Cr(VI) from marine environments [61,62]. The complete list of the main fungi, bacteria, and related species that have been described in the literature regarding bioremediation processes of heavy metals in marine sediments is reported in Table 1.

## 4. Marine Sediments Contaminated by Organic Pollutants: Approach Based on Bioremediation Mediated by Fungi and Bacteria

Organic contaminants affect marine biodiversity (e.g., marine mammals, intertidal and subtidal organisms, marine microorganisms, marine phanerogams, and algae), together with other organisms (such as seabirds), which exploit marine ecosystems for their vital needs, representing a concrete, actual, and increasing international problem that threatens not only the environment, but also human health [24,55,69]. Hydrocarbons, PAHs, and organic solvents are among the main organic pollutants in the sea (waters and sediments) [24,55].

In this dangerous and fragile scenario, the bioremediation of organic pollutants by fungi and bacteria represents a green, inexpensive, and efficient answer to the problem. The low impact on the marine ecosystem and biodiversity, in fact, is one of the most important advantages of this biotechnology. The latter exploits the fungal- and bacterial-mediated natural biodegradation of organic substances that are nutrients and food sources for these microorganisms. In the same way as for food, microbes can break hydrocarbon chains and aromatic rings through their metabolic pathways and enzymes [70,71]. Many researchers have shown the potentiality of microorganisms in bioremediation treatments of polluted marine environments [72,73,74]. However, studying the natural coexistence of different microorganisms in the marine environment suggests the possibility to employ microbial consortia to completely degrade the complex and multiple sources of contamination represented by petroleum hydrocarbons [24,69,75]. Indeed, each microbial species can efficiently biodegrade only some classes of organic pollutants due to its enzymes, whereas consortia of different species can be more aggressive, biodegrading many contaminants together [24].

### Fungi and Bacteria Involved in the Bioremediation of Organic Pollutants in Marine Sediments

The essential parameter for the application of bioremediation techniques to organic pollutants is the presence of vital microorganisms, mainly fungi and bacteria, in the contaminated area [23,26,27]. Many genera of aerobic bacteria, such as *Pseudomonas*, *Acinetobacter*, *Alcaligenes*, *Nocardia*, and *Rhodococcus*, can biodegrade petroleum hydrocarbons [24]. Among the *Pseudomonas* genus, in particular, it is well known that the *Pseudomonas fluorescens* group uses chrysene and benz[a]anthracene as a source of nutrients [76,77,78,79,80]. Similarly, *Rhodococcus* spp. are known to degrade hydrocarbons such as crude oil, diesel oil, and gasoline [81,82,83]. Crisafi et al. [84] reported the case of the Gulf of Taranto (Italy): after an oil spill event, they treated the seawater by using different bioremediation approaches. The results evidenced that, among all the methods employed, bioaugmentation with a hydrocarbonoclastic consortium composed of *Alcanivorax borkumensis*, *Alcanivorax dieselolei*, *Marinobacter hydrocarbonoclasticus*, *Cycloclasticus* sp. 78-ME, and *Thalassolituus oleivorans* degraded up to 79% of the hydrocarbons. In sediments from Genoa Harbour (Italy), Gallizia et al. [85] studied the best approach of bioremediation of organic polymers mediated by autochthonous microorganisms. They tested bioaugmentation (five different microorganisms), biostimulation (air supply), natural attenuation, and the coupling of bioaugmentation/biostimulation. After 60 days of bioaugmentation treatments, the microorganisms had multiplied, evidencing good metabolic activity. However, only the coupling of bioaugmentation with air insufflations produced the best response; bacterial densities and enzymatic activities increased, and sedimentary organic matter was degraded efficiently. Similarly, Dell’Anno et al. [86] carried out some bioremediation experiments on oxic and anoxic marine port sediments contaminated with hydrocarbons and showed changes in bacterial abundance and biodiversity. They indicated that higher temperatures increased bacterial abundance, diversity, and community composition in aerobic conditions, whilst the same parameters decreased in anaerobic conditions. In addition, the biodegradation rate was positively related to the bacterial richness. This finding suggests that bioremediation technologies could perform better in the hydrocarbon degradation of marine sediments if they supported high bacterial diversity and the selection of specific taxa. In addition, other recent reports have shown that halophilic bacteria and archaea can tolerate and survive in high-salt environments and can metabolise *n*-alkanes and PAHs, playing a central role in restoration plans of marine habitats contaminated with organic substances [87,88]. As discussed above, these technologies have the great potential to be low impact and are often the only strategies applicable on a large scale in marine environments [89,90].

Furthermore, several studies have shown that some fungi are characterised by the production of extracellular enzymes that make them excellent biodegraders of organic pollutants [74,91,92]. Microfungi mainly belonging to the genera *Aspergillus*, *Penicillium*, *Graphium*, *Neosartorya*, *Fusarium*, *Paecilomyces*, *Pseudallescheria*, and *Trichoderma* are the most well-known species with the capability to degrade a great variety of organic contaminants, such as petroleum hydrocarbons and PAHs [74,91,92,93,94,95]. Macrofungi are also known to biodegrade toxic organic substances, such as polychlorinated biphenyls (PCBs), PAHs, and hydrocarbons [92,96] in marine environments. Thanks to their different enzymes, fungi are able to break down complex organic compounds that are structurally similar to cellulose—called brown rotter fungi, such as *Lentinus ponderosus* O.K. Mill., *Gloeophyllum trabeum* (Persoon) Murrill, and *Serpula lacrymans* (Wulfen) J. Schröter, among others—or similar to lignin—called white rotter fungi, such as *Heterobasidion annosum* (Fr.) Bref. and *Phellinus punctatus* (P. Karst.) Pilát, among others. Some of the most important enzymes secreted and involved in the biodegradation of organic substances are: lignin peroxidase, manganese peroxidase, hydrogen peroxide-producing enzymes, and laccases [97,98]. Furthermore, recent studies have highlighted that the oyster mushroom *Pleurotus ostreatus* (Jacq.) P. Kumm, known for its ability to break hydrocarbons, can tolerate high-salt conditions and can be metabolically active in marine environments, thus representing an important alternative tool for the bioremediation of marine sediments [24]. Regarding yeast, a few reports have provided data on the potential bioremediation ability of this group of fungi and reported that *Candida*, *Pichia*, and *Yarrowia* are the most active genera in the degradation of oil hydrocarbons [99]. It is worth noting that fungi appear to have a higher biodegradation performance relative to bacteria, likely because fungi can degrade highly complex organic compounds, whereas bacteria degrade simpler substances with a low molecular weight [83,100]. Despite their high potentialities, there is a lack of information about the role of fungi in bioremediation of marine environments [91]. Researchers have characterised a fungal community able to degrade oil spills from Mediterranean marine (67 strains) and sediment (17 strains) samples. Among the isolates, they tested some species for the ability to degrade crude oil as a carbon source: *Aspergillus terreus* Thom, *Trichoderma harzianum* Rifai, and *Penicillium citreonigrum* Dierckx had the highest activity [91]. González-Abradelo et al. [101] studied the use of two halophilic fungi, *Aspergillus sydowii* (Bainier and Sartory) Thom and Church and *Aspergillus destruens* Zalar, F. Sklenar, S.W. Peterson and Hubka, for the elimination of PAHs and petroleum hydrocarbons in saline conditions. *A. sydowii* and *A. destruens* exploited benzo-α-pyrene and phenanthrene as nutrient sources and remediated up to 90% of both pollutants thanks to biodegradation and biosorption, respectively.

Recently, researchers have paid attention to the employment of microbial consortia of bacteria and fungi, which represent a more realistic simulation of environmental conditions [41,102]. These microorganisms, in fact, are well known (mainly in polluted soils) for their cooperation in environmental detoxification, plant growth promotion, and assisting phytoremediation [41,103]. However, as already mentioned, little is known about the application of microbial consortia of fungi and bacteria in marine sediment bioremediation [85,104]. The complete list of the main fungi, bacteria, and bioremediation techniques applied to marine sediments contaminated with organic pollutants is reported in Table 2.

## 5. Biosurfactants as Promising Tool for the Bioremediation of Marine Sediments

Several studies have shown the possibility to employ surfactants as a tool in remediation processes of heavy metals and/or organic pollutants in marine environments [126,127]. However, these chemicals are often toxic to the environment and can affect ecosystems. Recent biotechnological advances have evidenced a new surfactant production technology: biosurfactants, a natural and green alternative to chemical surfactants. They can be produced by fermentative processes using renewable resources, can be applied to many fields, and are characterised by low toxicity and high biodegradability [128,129,130,131]. Moreover, biosurfactants are a promising substitute because they can potentially be synthetised by a wide variety of microorganisms such as fungi and bacteria. They are a highly diverse group of structures [132] and represent an intriguing and alternative tool compared with the traditional bioremediation techniques in marine sediments. Despite their advantages, employing biosurfactants in bioremediation processes has not been widely disseminated, probably due to their high production costs. Biosurfactants derived from microorganisms generally have a lipid origin and are classified as: natural lipids, fatty acids, lipopolysaccharides, glycolipids, phospholipids, and lipopeptides [87,132,133]. Biosurfactants are amphipathic compounds characterised by a hydrophilic and a hydrophobic domain, which enable them to absorb hydrocarbons [132]. They are co-adjuvants in the degradation, recovery, and emulsification of oil substances and compounds [88]. Bacteria and fungi are the most important microorganisms able to produce biosurfactants. Both terrestrial and marine non-pathogenic species can synthetise them [134]. Bacterial species belonging to the genus *Pseudomonas*, including *Pseudomonas aeruginosa* and *P. fluorescens*, but also other species such as *Arthrobacter* spp., *Azotobacter chroococcum*, *Azotobacter vinelandii*, *Bacillus licheniformis*, and *Bacillus subtilis* [135,136], are well known as producers of biosurfactants. For example, biosurfactants such as sophorolipids are produced by *Torulopsis bombicola* J.F.T. Spencer, Gorin and A.P. Tulloch; *Starmerella apicola* (Hajsig) C.A. Rosa and Lachance; *Yarrowia lipolytica* (Wick., Kurtzman and Herman); *Candida tropicalis* (Castell.) Berkhout; *Moesziomyces antarcticus* (Goto, Sugiy., and Iizuka) Q.M. Wang, Begerow, F.Y. Bai, and Boekhout; and *Candida glabrata* (H.W. Anderson) S.A. Meyer and Yarrow [134,137,138]. Dell’Anno et al. [131] reported the best known and chemically characterised biosurfactants, including rhamnolipids produced by, for example, *P. aeruginosa* [139]; trehalose lipids by *Rhodococcus* sp., *Nocardia* sp., *Arthrobacter* sp., and *Mycobacterium* sp. [140]; cellobiolipids by *Ustilago maydis* (DC.) Corda [141]; sophorolipids by *Candida* sp. [142]; and mannosylerythriol lipids by *Moesziomyces antarcticus* [143]. Moreover, they discussed that other compounds produced by many bacteria and characterised by biosurfactant properties are lipo-peptides, such as surfactin and subtilisin, synthetised by *Bacillus subtilis*; lichenysin, synthesised by *B. licheniformis* and *B. subtilis* [144,145]; ornithine, synthesised by *Myroides* spp., *Pseudomonas* spp., *Thiobacillus* spp., *Agrobacterium* spp., and *Gluconobacter* spp. [146]; viscosin, synthesised by *Pseudomonas fluorescens* [147]; serrawettin, synthesised by *Serratia marcescens* [148]; fengycin, synthesised by *Bacillus* sp.; arthrofactin, synthesised by *Arthrobacter* sp.; and polymyxins, synthesised by *Bacillus polymyxa* and *Brevibacterium polymyxa* [136]. Hence, microorganisms play an important role as primary producers of biosurfactants, which can be exploited not only in bioremediation treatments of organic and inorganic toxic compounds in marine sediments [131,149,150] but also in many other fields such as medical applications, food production, cosmetic-related applications, and industrial processes [133].

Biosurfactants play a central role in biodegradation processes. They can improve the efficiency of the process by increasing the bioavailability of organic contaminants in the liquid phase by specific reactions such as solubilisation and micellisation [151]. This mechanism also favours the subsequent removal of contaminants by microorganisms via an integrated approach of bioremediation. In fact, it increases the attack surface for microbes. However, to date, it is not clear whether biosurfactants can also inhibit biodegradation, limiting the contact with contaminants. Hence, more studies should be carried out on the biodegradation rate of biosurfactants from microorganisms [151].

Concerning inorganic pollutants, biosurfactants can chemically attack heavy metals, desorbing, linking, and concentring metals to the sediment solid phase [152]. Cationic and anionic biosurfactants are the best known in metal bioremediation, exploiting the opposite charges and binding metals through polar heads [151,153].

Recently, within the family of biosurfactants compounds, bioemulsifiers have been recognised as a new group of substances employable for the remediation of marine polluted sediments [86]. These substances are a mixture of heteropolysaccharides, lipopolysaccharides, lipoproteins, and proteins [86,154]. However, Uzoigwe et al. [155] showed that bioemulsifiers are less effective in reducing surface tension compared with biosurfactants. Many marine microorganisms can produce bioemulsifiers [86], such as bacterial strains belonging to the *Myroides* genus, isolated after oil spill events [156], *Halomonas* sp. [157](Gutiérrez et al., 2007a), *Y. lipolytica* and *Antarctobacter* sp. [158], *Marinobacter arthrobacter* [159], and *Acinetobacter* sp. [153]. All these microbial strains represent a possible tool for the biorestoration of polluted sediments, but many other field studies should be conducted to verify and to improve their real applicability in situ.

Biosurfactants are very interesting biocompounds with high bioremediation potentiality: many microorganisms, including marine, synthetise them, and they could be employed not only in in situ sediment treatments, but also in ex situ treatment plants. Due to their chemical structure, they can speed up the degradation and inactivation process of pollutants, attacking contaminants effectively. Moreover, they can be exploited together with bacteria and fungi in a synergic integrated protocol to maximise results and remove multiple contaminants (e.g., metal and organic pollutants). Furthermore, as already discussed, the effectiveness and efficiency rate, the potential impacts, and the costs of bioremediation activities of marine sediments by biosurfactants need to be explored further by using field pilot experiments.

## 6. Conclusions

In the context of environmental remediation, the so called ‘traditional techniques’, physical–chemical methods, are characterised by high efficiency, but also by high costs and a high impact on ecosystems. On the contrary, biological methods are considered promising and environmentally friendly strategies, but they generally take longer, and it can be difficult to predict the yields of these technologies. As such, both techniques have advantages and disadvantages. However, some of the new bioremediation methods appear to be particularly promising, such as those involving the use of microorganisms. Microorganisms have been applied to remediate wastewater, soil, and solid waste, but also sediments. Fungi and bacteria are the most important in bioremediation of marine sediments, not only because of their high tolerance to organic/inorganic pollutants, but also because of their ability to actively degrade/inactivate a wide range of contaminants. In particular, fungi, due to their wide range of enzymes and production of secondary metabolites, are the most promising microorganisms in bioremediation of marine environments. Moreover, bacterial–fungal interactions and their synergistic bioremediation processes are important tools for the development of high-performance consortia that can effectively remediate many contaminants. Finally, biosurfactant application to marine sediments represents a new and interesting tool for the bioremediation of pollutants. Indeed, biosurfactants can be synthetised by many microorganisms (terrestrial and marine) and can be employed in integrated protocols of bioremediation. However, due to the heterogeneous and complex composition of marine sediments, it is evident that a single traditional or biological method cannot achieve the total remediation of pollutants. Currently, the most promising solutions consist of integrated approaches, meaning either the combined use of traditional methods and bioremediation, or bioremediation alone that uses selected consortia of organisms that act synergistically, such as fungi, bacteria, and biosurfactants.

## Figures and Tables

**Table 1:** List of the main autochthonous/allochthonous fungi, bacteria, and the related genera/species employable in marine sediment bioremediation processes of heavy metals (HMs).

Organisms	Genera/Species	HMs	Technologies	Autochthonous/ Allochthonous	References
Bacteria	*Desulfovibrio* spp.	Fe, U	Biostimulation	Autochthonous	[63]
	*Bacillus* spp.	Pb, Cu, Cr, Zn	Bioaccumlation		[64]
	*Micrococcus* spp.				
	*Kocuria* spp.				
	*Sporosarcina saromensis*	Cr (VI)	Biodegradation		[65]
Sulphate-Reducing Bacteria (SRB)	-	Fe, Cd, Cu, Pb	Biostimulant Ball (BSB)		[66]
Bacteria—*Phycisphaeraceae*	-	Cr, As	Biostimulation and biomobilisation		[21]
Bacteria—*Planctomycetaceae*,	-				
Bacteria—*Phyllobacteriaceae*	-				
Bacteria—*Desulfobacteraceae*	-	Cd	Stabilisation		
Bacteria—*Oceanospirillaceae*	-	Pb			
Bacteria—*Sinobacteraceae*	-				
Bacteria—*Flavobacteriaceae*	-				
	*Acidithiobacillus thiooxidans, Acidithiobacillus ferrooxidans, Leptospirillum ferrooxidans*, *Acidiphilium cryptum*	Cu, Zn, Cd, Hg, Ni, As, Pb, Cr	Bioaugmentation of consortia	Allochthonous	[1]
Bacteria	*Alcaligenes faecalis,* *Bacillus pumilus,* *Pseudomonas aeruginosa*, *Pseudomonas putida*, *Brevibacterium iodinium*	Hg	Volatilisation	Autochthonous	[40]
	*Pseudomonas aeruginosa*	Cd	Detoxification		
	*Bacillus pumilus, Bacillus iodinium, Pseudomonas Aeruginosa*	Pb	Detoxification		
	*Acidithiobacillus* spp.	Cu, Zn, Mn, Ni, Pb	Bioleaching		[52]
	*Acidithiobacillus thiooxidans*	Zn, Cd	Biomobilisation and re-suspension		[54]
	*Acidithiobacillus* spp.	Cr, Cu, Ni, Pb, Zn	Bioleaching		[11]
	*-*	Ni	Biosorption		[67]
	*Halomonas* sp., *Alcanivorax* sp., *Pseudoalteromonas* sp., *Epibacterium* sp., *Virgibacillus* sp.	As, Pb, Cd	Immobilisation by consortia		[55]
Fungi	*Aspergillus* sp.	Pb, As	Bioleaching		
	*Trichoderma viride*	Cr (VI), Cu	Bioaccumulation and biosorption	Autochthonous	[56]
	*Aspergillus niger*	Cd, Cu, Pb, Zn	Biomobilisation	Allochthonous	[68]
	*Penicillium expansum, Paecilomyces formosus*	Cu, Zn	Bioaccumulation by fungal-membrane consortia	Autochthonous	[10]
	*Penicillium brevicompactum, Mucor racemosus*	Cr, Ni, Mn			[57]
	*Cunninghamella elegans, Penicillium citrinum*	Cd, Cr, Zn, Sb			
	*Fusarium oxysporum, Cladosporium cladosporioides*	As, Cd			
	*Paradendryphiella salina*	Hg	Bioabsorption		[58]
	*Aspergillus spp.* (*A. flavus*, *A. niger*, *A. candidus*)	As	Bioaccumulation	Allochthonous	[59,60]
	*Yarrowia lipolytica*	Cr (VI)	Bioaccumulation		[61,62]

**Table 2:** List of the main microorganisms and bioremediation techniques of marine sediments contaminated by organic pollutants.

Organisms	Species	Organic Pollutants	Technologies	Autochthonous/ Allochthonous	References
Bacteria	*Cycloclasticus* sp.	PAHs	Bioaugmentation	Allochthonous	[105]
Bacteria—*Alphaproteobacteria*	-		Biostimulation	Autochthonous	[86]
Bacteria	*Alcanivorax* sp., *Thalassolituus* sp., *Cycloclasticus* sp.		Bioaugmentation		[106]
	-				[107]
	-				[108]
	-		Mixtures of inorganic nutrients and sand amendments		[109]
	*Bacillus methylotrophicus,* *Pseudomonas sihuiensis*	Petroleum hydrocarbons	Biodegradation		[27]
	*Thiohalophilus thiocyanatoxydans, Marinobacter* sp., *Planococcus* sp., *Thiobacillus* sp., *Erysipelothrix* sp., *Clostridium* sp., *Halothiobacillus* sp.*, Halobacillus* sp., *Tenacibaculum* sp., *Isoprenicola* sp.	Total petroleum hydrocarbons (TPHs)	Biostimulation and bioaugmentation	Allochthonous	[110]
Bacteria	-	Phenanthrene (PHE), PAHs	Biostimulation and bioaugmentation	Autochthonous	[16]
	-	Hydrocarbons	Biostimulation		[111]
	*Pseudomonas* sp.	PAHs	Bioreactor		[112]
	*Acinetobacter calcium acetate, Pseudomonas putida, Salfobacillus* sp.		Bioremediation using zeolite carrier		[113]
	-	TPHs	Biostimulation		[114]
	-	Crude oil			[115]
	-	PAHs			[116]
	-				[117]
	*Bacillus* sp.	Emerging organic contaminants	Biodegradation		[118]
	-	TPHs	Biostimulation and bioaugmentation	Autochthonous	[119]
	*Marinobacter* sp., *Pseudomonas* sp*., Halomonas* sp*., Hahella* sp*., Alcanivorax* sp.	Oil			[120]
	*Erythrobacter* sp.*, Alcaligenes* sp.	Benzo(a)pyrene and fluoranthene	Bioaccumulation and biodegradation		[121]
	-	PAHs	Biostimulation		[122]
Fungi	*Aspergillus* sp.	PAHs	Bioaugmentation		[55]
	*Aspergillus sydowii, Aspergillus destruens*		Biodegradation	Allochthonous	[101]
	*Pleurotus ostreatus*	TPEs	Bioaugmentation		[123]
Fungi	*Lambertella* sp.	TPHs		Autochthonous	[124]
Fungi	*Saccharomyces cerevisiae,* *Scopulariopsis* sp.*,* *Bulgaria* sp./*Cyttaria* sp.	Total Petroleum Hydrocarbons (TPHs)	Biostimulation and bioaugmentation	Allochthonous	[110]
Co-coltures of fungi and bacteria	-	Petroleum hydrocarbons	Bioaugmentation		[104]
	*Bacillus subtilis, Bacillus licheniformis, Pseudomonas* *putida, Lactobacillus helveticus, Lactococcus lactis,* *Trichoderma reesei, Trichoderma harzianum, Phanerochaete* *chrysosporium, Nitrosomonas sp., Acinetobacter genospecies, Arthrobacter* sp.	Organic polymers	Bioaugmentation and biostimulation	Autochthonous	[85]
	-	PAHs	Bioaugmentation	Allochthonous	[125]

## Data Availability

Not applicable.

## Glossary

**Technology**	**Application**
Biostimulation	Stimulation of the metabolic activities of native microbial communities by the inoculation of specific nutrients.
Bioaccumulation	Active transport and storage of metals within microbial cells due to metabolism (and/or low molecular weight chelating agents/proteins) (bioimmobilization approach).
Biodegradation	Breakdown of organic substances by microorganisms.
Biomobilization	Exploitation of organic acids and metabolites produced by microbes to mobilize metals into a liquid medium by chemical reactions.
Bioimmobilization/Stabilization	Exploitation of microorganisms to inactivate metal toxicity through microbial capability, reducing metal mobility, bioavailability, and toxicity without completely removing them from substrates.
Bioaugmentation	Microbial strain inoculation in the substrates to be remediated.
Volatilization	Process in which certain species of microorganisms transform metallic compounds into gaseous molecules by biomethylation.
Detoxification mediated by microbes	Removal of toxic substances from the environment by microorganisms.
Bioleaching	Exploitation of biological redox reactions, acid secretions, and pH changes to transform metals into soluble states (biomobilization approach).
Biosorption	Association of soluble HMs with the cell wall or the membrane surface of microorganisms through many mechanisms such as complexation, chelation, reduction, and precipitation (bioimmobilization approach).
Bioreactor	Machinery capable of providing a suitable environment for the mass growth of biological organisms.

## References

[1] Beolchini F., Dell’Anno A., De Propris L., Ubaldini S., Cerrone F., Danovaro R. (2009). Auto- and heterotrophic acidophilic bacteria enhance the bioremediation efficiency of sediments contaminated by heavy metals. Chemosphere.

[2] Sollecito F., Vitone C., Miccoli D., Plötze M., Puzrin A.M., Cotecchia F. (2019). Marine Sediments from a Contaminated Site: Geotechnical Properties and Chemo-Mechanical Coupling Processes. Geosciences.

[3] Katsiri A., Pantazidou M., Damikouka I., Kontogiorgi C., Tringali A. (2009). Disposal options for dredged marine sediments based on physicochemical and toxicological characterization. Glob. NEST J..

[4] Harrington J., Murphy J., Coleman M., Jordan D., Debuigne T., Szacsuri G. (2016). Economic modelling of the management of dredged marine sediments. Geol. Geophys. Environ..

[5] Harrington J., Murphy J., Coleman M., Jordan D., Szacsuri G. (2016). Financial modelling and analysis of the management of dredged marine sediments—Development of a decision support tool. J. Shipp. Trade.

[6] Manap N., Voulvoulis N. (2015). Environmental management for dredging sediments—The requirement of developing nations. J. Environ. Manag..

[7] Mat-Shayuti M.S., Ya T.M.Y.S.T., Abdullah M.Z., Khamaruddin P.N.F.M., Othman N.H. (2019). Progress in ultrasonic oil-contaminated sand cleaning: A fundamental review. Environ. Sci. Pollut. Res..

[8] Sprovieri M., Feo M.L., Prevedello L., Manta D.S., Sammartino S., Tamburrino S., Marsella E. (2007). Heavy metals, polycyclic aromatic hydrocarbons and polychlorinated biphenyls in surface sediments of the Naples harbour (southern Italy). Chemosphere.

[9] Mattei P., Cincinelli A., Martellini T., Natalini R., Pascale E., Renella G. (2016). Reclamation of river dredged sediments polluted by PAHs by co-composting with green waste. Sci. Total Environ..

[10] Cecchi G., Cutroneo L., Di Piazza S., Vagge G., Capello M., Zotti M. (2019). From waste to resource: Mycoremediation of contaminated marine sediments in the SEDITERRA Project. J. Soils Sediments.

[11] Seidel H., Löser C., Zehnsdorf A., Hoffmann P., Schmerold R. (2004). Bioremediation Process for Sediments Contaminated by Heavy Metals: Feasibility Study on a Pilot Scale. Environ. Sci. Technol..

[12] Lee B.D., Hosomi M. (2000). Ethanol washing of PAH-contaminated soil and Fenton oxidation of washing solution. J. Mater. Cycles Waste.

[13] Sun Y., Ji L., Wang W., Wang X., Wu J., Li H., Guo H. (2009). Simultaneous Removal of Polycyclic Aromatic Hydrocarbons and Copper from Soils using Ethyl Lactate-Amended EDDS Solution. J. Environ. Qual..

[14] Yap C.L., Gan S., Ng H.K. (2012). Evaluation of solubility of polycyclic aromatic hydrocarbons in ethyl lactate/water versus ethanol/water mixtures for contaminated soil remediation applications. J. Environ. Sci..

[15] Ferraro A., Fabbricino M., Van Hullebusch E.D., Esposito G. (2017). Investigation of different ethylenediamine-N,N′-disuccinic acid-enhanced washing configurations for remediation of a Cu-contaminated soil: Process kinetics and efficiency comparison between single-stage and multi-stage configurations. Environ. Sci. Pollut. Res..

[16] Bianco F., Monteverde G., Race M., Papirio S., Esposito G. (2020). Comparing performances, costs and energy balance of ex situ remediation processes for PAH-contaminated marine sediments. Environ. Sci. Pollut. Res..

[17] Smith M.T., Berruti F., Mehrotra A.K. (2001). Thermal Desorption Treatment of Contaminated Soils in a Novel Batch Thermal Reactor. Ind. Eng. Chem. Res..

[18] Falciglia P.P., Malarbì D., Roccaro P., Vagliasindi F.G. (2020). Innovative thermal and physico-chemical treatments for the clean-up of marine sediments dredged from the Augusta Bay (Southern Italy). Reg. Stud. Mar. Sci..

[19] Lofrano G., Libralato G., Minetto D., De Gisi S., Todaro F., Conte B., Calabrò D., Quatraro L., Notarnicola M. (2017). In situ remediation of contaminated marinesediment: An overview. Environ. Sci. Pollut. Res..

[20] Rocchetti L., Beolchini F., Hallberg K.B., Johnson D.B., Dell’Anno A. (2012). Effects of prokaryotic diversity changes on hydrocarbon degradation rates and metal partitioning during bioremediation of contaminated anoxic marine sediments. Mar. Pollut. Bull..

[21] Fonti V., Beolchini F., Rocchetti L., Dell’Anno A. (2015). Bioremediation of contaminated marine sediments can enhance metal mobility due to changes of bacterial diversity. Water Res..

[22] Chang C.-Y., Chen S.-Y., Klipkhayai P., Chiemchaisri C. (2019). Bioleaching of heavy metals from harbor sediment using sulfur-oxidizing microflora acclimated from native sediment and exogenous soil. Environ. Sci. Pollut. Res..

[23] Adams G.O., Fufeyin P.T., Okoro S.E., Ehinomen I. (2020). Bioremediation, Biostimulation and Bioaugmention: A Review. Int. J. Environ. Bioremediat. Biodegrad..

[24] Kumar V., Kumar M., Prasad R. (2018). Microbial Action on Hydrocarbons. Microbial Action on Hydrocarbons.

[25] Kumar V., Dwivedi S.K. (2021). Mycoremediation of heavy metals: Processes, mechanisms, and affecting factors. Environ. Sci. Pollut. Res..

[26] Verma J.P., Jaiswal D.K. (2016). Book Review: Advances in Biodegradation and Bioremediation of Industrial Waste. Front. Microbiol..

[27] Pereira E., Napp A., Allebrandt S., Barbosa R., Reuwsaat J., Lopes W., Kmetzsch L., Staats C.C., Schrank A., Dallegrave A. (2019). Biodegradation of aliphatic and polycyclic aromatic hydrocarbons in seawater by autochthonous microorganisms. Int. Biodeterior. Biodegrad..

[28] Peeples T.L. (2014). Bioremediation Using Extremophiles. Microbial Biodegradation and Bioremediation.

[29] Spina F., Cecchi G., Torres A.Y.L., Pecoraro L., Russo F., Wu B., Cai L., Liu X.Z., Tosi S., Varese G.C. (2018). Fungi as a toolbox for sustainable bioremediation of pesticides in soil and water. Plant Bios.

[30] Giovanella P., Vieira G.A., Otero I., Pellizzer E.P., Fontes B.D.J., Sette L.D. (2020). Metal and organic pollutants bioremediation by extremophile microorganisms. J. Hazard. Mater..

[31] Gadd G.M. (2007). Geomycology: Biogeochemical transformations of rocks, minerals, metals and radionuclides by fungi, bioweathering and bioremediation. Mycol. Res..

[32] Ramos J.-L., Marqués S., Van Dillewijn P., Espinosa-Urgel M., Segura A., Duque E., Krell T., Ramos-González M.-I., Bursakov S., Roca A. (2011). Laboratory research aimed at closing the gaps in microbial bioremediation. Trends Biotechnol..

[33] Fomina M., Gadd G.M. (2014). Biosorption: Current perspectives on concept, definition and application. Bioresour. Technol..

[34] Kumar B.L., Gopal D.V.R.S. (2015). Effective role of indigenous microorganisms for sustainable environment. 3 Biotech.

[35] Raddadi N., Cherif A., Daffonchio D., Neifar M., Fava F. (2015). Biotechnological applications of extremophiles, extremozymes and extremolytes. Appl. Microbiol. Biotechnol..

[36] Gadd G.M., Rhee Y.J., Stephenson K., Wei Z. (2012). Geomycology: Metals, actinides and biominerals. Environ. Microbiol. Rep..

[37] Villela H.D., Peixoto R.S., Soriano A.U., Carmo F.L.D. (2019). Microbial bioremediation of oil contaminated seawater: A survey of patent deposits and the characterization of the top genera applied. Sci. Total. Environ..

[38] Giovanella P., Cabral L., Costa A.P., Camargo F.A.D.O., Gianello C., Bento F.M. (2017). Metal resistance mechanisms in Gram-negative bacteria and their potential to remove Hg in the presence of other metals. Ecotoxicol. Environ. Saf..

[39] Peng W., Li X., Xiao S., Fan W. (2018). Review of remediation technologies for sediments contaminated by heavy metals. J. Soils Sediments.

[40] De J., Ramaiah N., Vardanyan L. (2008). Detoxification of Toxic Heavy Metals by Marine Bacteria Highly Resistant to Mercury. Mar. Biotechnol..

[41] Ceci A., Pinzari F., Russo F., Persiani A.M., Gadd G.M. (2019). Roles of saprotrophic fungi in biodegradation or transformation of organic and inorganic pollutants in co-contaminated sites. Appl. Microbiol. Biotechnol..

[42] Seidel H., Ondrushka J., Morgenstem P., Stottmeister U. (1998). Bioleaching of heavy metals from contaminated aquatic sediments using indigenous sulfur-oxidizing bacteria: A feasibility study. Water Sci. Technol..

[43] De Oliveira D.M., Sobral L.G., Olson G.J., Olson S.B. (2014). Acid leaching of a copper ore by sulphur-oxidizing microorganisms. Hydrometallurgy.

[44] Castro L., Blázquez M.L., González F., Muñoz J.A. (2020). Bioleaching of Phosphate Minerals Using *Aspergillus niger*: Recovery of Copper and Rare Earth Elements. Metals.

[45] Tabak H.H., Lens P., Van Hullebusch E.D., Dejonghe W. (2005). Developments in Bioremediation of Soils and Sediments Polluted with Metals and Radionuclides—1. Microbial Processes and Mechanisms Affecting Bioremediation of Metal Contamination and Influencing Metal Toxicity and Transport. Rev. Environ. Sci. Bio/Technol..

[46] Ahemad M. (2014). Bacterial mechanisms for Cr(VI) resistance and reduction: An overview and recent advances. Folia Microbiol..

[47] Tsezos M. (2009). Metal—Microbes Interactions: Beyond Environmental Protection. Adv. Mater. Res..

[48] Bosecker K. (1997). Bioleaching: Metal solubilization by microorganisms. FEMS Microbiol. Rev..

[49] Chaturvedi A.D., Pal D., Penta S., Kumar A. (2015). Ecotoxic heavy metals transformation by bacteria and fungi in aquatic ecosystem. World J. Microbiol. Biotechnol..

[50] Chen S.-Y., Lin J.-G. (2001). Bioleaching of heavy metals from sediment: Significance of pH. Chemosphere.

[51] Chen S.-Y., Lin J.-G. (2001). Effect of substrate concentration on bioleaching of metal-contaminated sediment. J. Hazard. Mater..

[52] Chen S.-Y., Lin J.-G. (2004). Bioleaching of heavy metals from contaminated sediment by indigenous sulfur-oxidizing bacteria in an air-lift bioreactor: Effects of sulfur concentration. Water Res..

[53] Zhang Y., Zhang S., Zhao D., Ni Y., Wang W., Yan L. (2019). Complete Genome Sequence of Acidithiobacillus ferrooxidans YNTRS-40, a Strain of the Ferrous Iron- and Sulfur-Oxidizing Acidophile. Microorganisms.

[54] Lors C., Tiffreau C., Laboudigue A. (2004). Effects of bacterial activities on the release of heavy metals from contaminated dredged sediments. Chemosphere.

[55] Dell’Anno F., Brunet C., Van Zyl L.J., Trindade M., Golyshin P.N., Dell’Anno A., Ianora A., Sansone C. (2020). Degradation of Hydrocarbons and Heavy Metal Reduction by Marine Bacteria in Highly Contaminated Sediments. Microorganisms.

[56] El-Kassas H.Y., El-Taher E.M. (2009). Optimization of batch process parameters by response surface methodology for mycoremedi-ation of chrome-VI by a chromium resistant strain of marine Trichoderma viride. Am. Eurasian J. Agric. Environ. Sci..

[57] Cecchi G., Vagge G., Cutroneo L., Greco G., Di Piazza S., Faga M., Zotti M., Capello M. (2019). Fungi as potential tool for polluted port sediment remediation. Environ. Sci. Pollut. Res..

[58] Panseriya H.Z., Gosai H.B., Sachaniya B.K., Vala A.K., Dave B.P. (2019). Marine Microbial Mettle for Heavy Metal Bioremediation: A Perception. Monitoring Artificial Materials and Microbes in Marine Ecosystems: Interactions and Assessment Methods.

[59] Vala A.K. (2010). Tolerance and removal of arsenic by a facultative marine fungus Aspergillus candidus. Bioresour. Technol..

[60] Vala A.K., Sachaniya B., Dave B.P. (2018). Marine-Derived Fungi: Promising Candidates for Enhanced Bioremediation. Nanomaterial Biointeractions at the Cellular, Organismal and System Levels.

[61] Rao A., Bankar A., Kumar A.R., Gosavi S., Zinjarde S. (2013). Removal of hexavalent chromium ions by Yarrowia lipolytica cells modified with phyto-inspired Fe0/Fe3O4 nanoparticles. J. Contam. Hydrol..

[62] Imandi S.B., Chinthala R., Saka S., Vechalapu R.R., Nalla K.K. (2014). Optimization of chromium biosorption in aqueous solution by marine yeast biomass of *Yarrowia lipolytica* using Doehlert experimental design. African J. Biotechnol..

[63] Cardenas E., Wu W.-M., Leigh M.B., Carley J., Carroll S., Gentry T., Luo J., Watson D., Gu B., Ginder-Vogel M. (2008). Microbial Communities in Contaminated Sediments, Associated with Bioremediation of Uranium to Submicromolar Levels. Appl. Environ. Microbiol..

[64] Jroundi F., Martinez-Ruiz F., Merroun M.L., Gonzalez-Muñoz M.T. (2020). Exploring bacterial community composition in Medi-terranean deep-sea sediments and their role in heavy metal accumulation. Sci. Total Environ..

[65] Ran Z., Bi W., Cai Q., Min L., Dong H., Dong Bei G., Juan W., Chun F. (2016). Bioremediation of hexavalent chromium pollu-tion by Sporosarcina saromensis M52 isolated from offshore sediments in Xiamen, China. Biomed. Environ. Sci..

[66] Subha B., Song Y.C., Woo J.H. (2017). Bioremediation of contaminated coastal sediment: Optimization of slow release biostimulant ball using response surface methodology (RSM) and stabilization of metals from contaminated sediment. Mar. Pollut. Bull..

[67] Pringault O., Viret H., Duran R. (2010). Influence of microorganisms on the removal of nickel in tropical marine sediments (New Caledonia). Mar. Pollut. Bull..

[68] Zeng X., Twardowska I., Wei S., Sun L., Wang J., Zhu J., Cai J. (2015). Removal of trace metals and improvement of dredged sediment dewaterability by bioleaching combined with Fenton-like reaction. J. Hazard. Mater..

[69] Zhang M., He P., Qiao G., Wang J., Huang J., Yuan X., Li Q. (2019). Distribution, sources, and risk assessment of polycyclic aromatic hydrocarbons (PAHs) in surface sediments of the Subei Shoal, China. Mar. Pollut. Bull..

[70] Finley S.D., Broadbelt L.J., Hatzimanikatis V. (2010). In silico feasibility of novel biodegradation pathways for 1,2,4-trichlorobenzene. BMC Syst. Biol..

[71] Loss E.M.O., Yu J.-H. (2018). Bioremediation and microbial metabolism of benzo(a)pyrene. Mol. Microbiol..

[72] Mapelli F., Scoma A., Michoud G., Aulenta F., Boon N., Borin S., Kalogerakis N., Daffonchio D. (2017). Biotechnologies for Marine Oil Spill Cleanup: Indissoluble Ties with Microorganisms. Trends Biotechnol..

[73] Crini G., Lichtfouse E. (2018). Wastewater Treatment: An Overview. Nanosensors for Environment, Food and Agriculture Vol. 1.

[74] Greco G., Di Piazza S., Cecchi G., Cutroneo L., Capello M., Zotti M. (2019). Mycoremediation of Oily Slime Containing a Polycyclic Aromatic Hydrocarbon Mixture. Waste Biomass Valorization.

[75] Brune K.D., Bayer T.S. (2012). Engineering microbial consortia to enhance biomining and bioremediation. Front. Microbiol..

[76] Caldini G., Cenci G., Manenti R., Morozzi G. (1995). The ability of an environmental isolate of Pseudomonas fluorescens to utilize chrysene and other four-ring polynuclear aromatic hydrocarbons. Appl. Microbiol. Biotechnol..

[77] Husain S. (2008). Identification of pyrene-degradation pathways: Bench-scale studies usingPseudomonas fluorescens 29L. Remediat. J..

[78] Ma J., Xu L., Jia L. (2013). Characterization of pyrene degradation by Pseudomonas sp. strain Jpyr-1 isolated from active sewage sludge. Bioresour. Technol..

[79] Sangkharak K., Choonut A., Rakkan T., Prasertsan P. (2020). The Degradation of Phenanthrene, Pyrene, and Fluoranthene and Its Conversion into Medium-Chain-Length Polyhydroxyalkanoate by Novel Polycyclic Aromatic Hydrocarbon-Degrading Bacteria. Curr. Microbiol..

[80] Nzila A., Musa M.M. (2020). Current Status of and Future Perspectives in Bacterial Degradation of Benzo[a]pyrene. Int. J. Environ. Res. Public Health.

[81] Kuyukina M.S., Ivshina I.B. (2010). Application of Rhodococcus in Bioremediation of Contaminated Environments. Benef. Microorg. Food Nutraceuticals.

[82] Hackbusch S., Noirungsee N., Viamonte J., Sun X., Bubenheim P., Kostka J.E., Müller R., Liese A. (2020). Influence of pressure and dispersant on oil biodegradation by a newly isolated Rhodococcus strain from deep-sea sediments of the gulf of Mexico. Mar. Pollut. Bull..

[83] Peng R.-H., Xiong A.-S., Xue Y., Fu X.-Y., Gao F., Zhao W., Tian Y.-S., Yao Q.-H. (2008). Microbial biodegradation of polyaromatic hydrocarbons. FEMS Microbiol. Rev..

[84] Crisafi F., Genovese M., Smedile F., Russo D., Catalfamo M., Yakimov M., Giuliano L., Denaro R. (2016). Bioremediation technologies for polluted seawater sampled after an oil-spill in Taranto Gulf (Italy): A comparison of biostimulation, bioaugmentation and use of a washing agent in microcosm studies. Mar. Pollut. Bull..

[85] Gallizia I., Vezzulli L., Fabiano M. (2005). Evaluation of different bioremediation protocols to enhance decomposition of organic polymers in harbour sediments. Biodegradation.

[86] Dell’Anno A., Beolchini F., Rocchetti L., Luna G.M., Danovaro R. (2012). High bacterial biodiversity increases degradation performance of hydrocarbons during bioremediation of contaminated harbor marine sediments. Environ. Pollut..

[87] Paniagua-Michel J., Rosales A. (2015). Marine bioremediation—A sustainable biotechnology of petroleum hydrocarbons biodegra-dation in coastal and marine environments. J. Bioremed. Biodegrad..

[88] Paniagua-Michel J., Fathepure B.Z. (2018). Microbial Consortia and Biodegradation of Petroleum Hydrocarbons in Marine Environments. Microbial Action on Hydrocarbons.

[89] Snape I., Riddle M.J., Stark J., Cole C.M., King C., Duquesne S., Gore D.B. (2001). Management and remediation of contaminated sites at Casey Station, Antarctica. Polar Rec..

[90] Hassanshahian M., Emtiazi G., Caruso G., Cappello S. (2014). Bioremediation (bioaugmentation/biostimulation) trials of oil polluted seawater: A mesocosm simulation study. Mar. Environ. Res..

[91] Bovio E., Gnavi G., Prigione V., Spina F., Denaro R., Yakimov M., Calogero R., Crisafi F., Varese G.C. (2017). The culturable mycobiota of a Mediterranean marine site after an oil spill: Isolation, identification and potential application in bioremediation. Sci. Total Environ..

[92] Sánchez C. (2020). Fungal potential for the degradation of petroleum-based polymers: An overview of macro- and microplastics biodegradation. Biotechnol. Adv..

[93] Al Tamie M.S. (2014). Effect of Salinity on the Fungal Occurance in Al-Shega Area at Al-Qassim, Saudi Arabia. Res. J. Microbiol..

[94] Daccò C., Nicola L., Temporiti M.E.E., Mannucci B., Corana F., Carpani G., Tosi S. (2020). Trichoderma: Evaluation of Its Degrading Abilities for the Bioremediation of Hydrocarbon Complex Mixtures. Appl. Sci..

[95] Venice F., Davolos D., Spina F., Poli A., Prigione V., Varese G., Ghignone S. (2020). Genome Sequence of *Trichoderma lixii* MUT3171, A Promising Strain for Mycoremediation of PAH-Contaminated Sites. Microorganisms.

[96] Baniasadi M., Mousavi S.M. (2018). A Comprehensive Review on the Bioremediation of Oil Spills. Microbial Action on Hydrocarbons.

[97] Rhodes C.J. (2014). Mycoremediation (bioremediation with fungi)—Growing mushrooms to clean the earth. Chem. Speciat. Bioavailab..

[98] Upadhyay P., Shrivastava R., Agrawal P.K. (2016). Bioprospecting and biotechnological applications of fungal laccase. 3 Biotech.

[99] Zinjarde S., Apte M., Mohite P., Kumar A.R. (2014). Yarrowia lipolytica and pollutants: Interactions and applications. Biotechnol. Adv..

[100] Fernández-Luqueño F., Valenzuela-Encinas C., Marsch R., Martínez-Suárez C., Vázquez-Núñez E., Dendooven L. (2011). Microbial communities to mitigate contamination of PAHs in soil—possibilities and challenges: A review. Environ. Sci. Pollut. Res..

[101] González-Abradelo D., Pérez-Llano Y., Peidro-Guzmán H., Sánchez M.D.R., Folch-Mallol J., Aranda E., Vaidyanathan V.K., Cabana H., Gunde-Cimerman N., Batista-García R.A. (2019). First demonstration that ascomycetous halophilic fungi (Aspergillus sydowii and Aspergillus destruens) are useful in xenobiotic mycoremediation under high salinity conditions. Bioresour. Technol..

[102] Alisi C., Musella R., Tasso F., Ubaldi C., Manzo S., Cremisini C., Sprocati A.R. (2009). Bioremediation of diesel oil in a co-contaminated soil by bioaugmentation with a microbial formula tailored with native strains selected for heavy metals resistance. Sci. Total. Environ..

[103] Rosatto S., Roccotiello E., Di Piazza S., Cecchi G., Greco G., Zotti M., Vezzulli L., Mariotti M. (2019). Rhizosphere response to nickel in a facultative hyperaccumulator. Chemosphere.

[104] Mikesková H., Novotný Č., Svobodová K. (2012). Interspecific interactions in mixed microbial cultures in a biodegradation per-spective. Appl. Microbiol. Biotechnol..

[105] Miyasaka T., Asami H., Watanabe K. (2006). Impacts of Bioremediation Schemes on Bacterial Population in Naphthalene-Contaminated Marine Sediments. Biodegradation.

[106] McKew B.A., Coulon F., Yakimov M.M., Denaro R., Genovese M., Smith C.J., Osborn A.M., Timmis K.N., McGenity T.J. (2007). Efficacy of intervention strategies for bioremediation of crude oil in marine systems and effects on indigenous hydrocarbonoclastic bacteria. Environ. Microbiol..

[107] Syakti A.D., Mazzella N., Nerini D., Guiliano M., Bertrand J., Doumenq P. (2006). Phospholipid fatty acids of a marine sedimentary microbial community in a laboratory microcosm: Responses to petroleum hydrocarbon contamination. Org. Geochem..

[108] Wang Y., Tam N. (2011). Microbial community dynamics and biodegradation of polycyclic aromatic hydrocarbons in polluted marine sediments in Hong Kong. Mar. Pollut. Bull..

[109] Beolchini F., Rocchetti L., Regoli F., Dell’Anno A. (2010). Bioremediation of marine sediments contaminated by hydrocarbons: Experimental analysis and kinetic modeling. J. Hazard. Mater..

[110] Doni S., Macci C., Martinelli C., Iannelli R., Brignoli P., Lampis S., Andreolli M., Vallini G., Masciandaro G. (2018). Combination of sediment washing and bioactivators as a potential strategy for dredged marine sediment recovery. Ecol. Eng..

[111] Bellagamba M., Viggi C.C., Ademollo N., Rossetti S., Aulenta F. (2017). Electrolysis-driven bioremediation of crude oil-contaminated marine sediments. New Biotechnol..

[112] Vosoughi M., Moslehi P., Aalemzadeh I. (2005). Some investigation on bioremediation of sediment in Persian Gulf Coast. Int. J. Eng..

[113] Wang C., He S., Zou Y., Liu J., Zhao R., Yin X., Zhang H., Li Y. (2020). Quantitative evaluation of in-situ bioremediation of compound pollution of oil and heavy metal in sediments from the Bohai Sea, China. Mar. Pollut. Bull..

[114] Zhang Z., Lo I.M., Yan D.Y. (2015). An integrated bioremediation process for petroleum hydrocarbons removal and odor mitigation from contaminated marine sediment. Water Res..

[115] Hamdan H.Z., Salam D.A. (2020). Microbial community evolution during the aerobic biodegradation of petroleum hydrocarbons in marine sediment microcosms: Effect of biostimulation and seasonal variations. Environ. Pollut..

[116] Bianco F., Race M., Papirio S., Esposito G. (2020). Removal of polycyclic aromatic hydrocarbons during anaerobic biostimulation of marine sediments. Sci. Total Environ..

[117] Sakaya K., Salam D.A., Campo P. (2019). Assessment of crude oil bioremediation potential of seawater and sediments from the shore of Lebanon in laboratory microcosms. Sci. Total Environ..

[118] Azaroff A., Monperrus M., Miossec C., Gassie C., Guyoneaud R. (2021). Microbial degradation of hydrophobic emerging contaminants from marine sediment slurries (Capbreton Canyon) to pure bacterial strain. J. Hazard. Mater..

[119] Gouveia V., Almeida C.M.R., Almeida T., Teixeira C., Mucha A.P. (2018). Indigenous microbial communities along the NW Portuguese Coast: Potential for hydrocarbons degradation and relation with sediment contamination. Mar. Pollut. Bull..

[120] Abed R.M., Al-Sabahi J., Al-Maqrashi F., Al-Habsi A., Al-Hinai M. (2014). Characterization of hydrocarbon-degrading bacteria isolated from oil-contaminated sediments in the Sultanate of Oman and evaluation of bioaugmentation and biostimulation approaches in microcosm experiments. Int. Biodeterior. Biodegrad..

[121] Kahla O., Ben Garali S.M., Karray F., Ben Abdallah M., Kallel N., Mhiri N., Zaghden H., Barhoumi B., Pringault O., Quéméneur M. (2021). Efficiency of benthic diatom-associated bacteria in the removal of benzo(a)pyrene and fluoranthene. Sci. Total. Environ..

[122] Louvado A., Gomes N., Simões M., Almeida A., Cleary D., Cunha A. (2015). Polycyclic aromatic hydrocarbons in deep sea sediments: Microbe–pollutant interactions in a remote environment. Sci. Total. Environ..

[123] Siracusa G., Becarelli S., Chicca I., Condino F., de Lima Silva M.R., Ruffini Castiglione M., Petroni G., Munz G., Lorenzi R., Di Gregorio S. Recovering of dredged sediments contaminated by total petroleum hydrocarbon to productive soils: The mycoremediation approach in the Bioresnova project. Proceedings of the X International Symposium On Sanitary And Environmental Engi-neering, SIDISA.

[124] Becarelli S., Chicca I., Siracusa G., La China S., Gentini A., Lorenzi R., Munz G., Petroni G., Levin D.B., Di Gregorio S. (2019). Hydrocarbonoclastic Ascomycetes to enhance co-composting of total petroleum hydrocarbon (TPH) contaminated dredged sediments and lignocellulosic matrices. New Biotechnol..

[125] Bucens P., Seech A., Marvan I. (1996). Pilot-scale demonstration of DARAMEND enhanced bioremediation of sediment contami-nated with polycyclic aromatic hydrocarbons in Hamilton Harbour. Water Qual. Res. J..

[126] Develter D.W.G., Lauryssen L.M.L. (2010). Properties and industrial applications of sophorolipids. Eur. J. Lipid Sci. Technol..

[127] Franzetti A., Gandolfi I., Bestetti G., Smyth T.J.P., Banat I.M. (2010). Production and applications of trehalose lipid biosurfactants. Eur. J. Lipid Sci. Technol..

[128] Cameotra S.S., Makkar R.S. (2010). Biosurfactant-enhanced bioremediation of hydrophobic pollutants. Pure Appl. Chem..

[129] Hazra C., Kundu D., Ghosh P., Joshi S., Dandi N., Chaudhari A. (2010). Screening and identification of Pseudomonas aeruginosa AB4 for improved production, characterization and application of a glycolipid biosurfactant using low-cost agro-based raw materials. J. Chem. Technol. Biotechnol..

[130] Zhao F., Shi R., Cui Q., Han S., Dong H., Zhang Y. (2017). Biosurfactant production under diverse conditions by two kinds of biosurfactant-producing bacteria for microbial enhanced oil recovery. J. Pet. Sci. Eng..

[131] Dell’Anno F., Sansone C., Ianora A. (2018). Biosurfactant-induced remediation of contaminated marine sediments: Current knowledge and future perspectives. Mar. Environ. Res..

[132] Lee D.W., Lee H., Kwon B.-O., Khim J.S., Yim U.H., Kim B.S., Kim J.-J. (2018). Biosurfactant-assisted bioremediation of crude oil by indigenous bacteria isolated from Taean beach sediment. Environ. Pollut..

[133] Kubicki S., Bollinger A., Katzke N., Jaeger K.E., Loeschcke A., Thies S. (2019). Marine biosurfactants: Biosynthesis, structural di-versity and biotechnological applications. Marine Drugs.

[134] Tripathi L., Irorere V.U., Marchant R., Banat I.M. (2018). Marine derived biosurfactants: A vast potential future resource. Biotechnol. Lett..

[135] Elliot R., Singhal N., Swift S. (2010). Surfactants and Bacterial Bioremediation of Polycyclic Aromatic Hydrocarbon Contaminated Soil—Unlocking the Targets. Crit. Rev. Environ. Sci. Technol..

[136] Shekhar S., Sundaramanickam A., Balasubramanian T. (2015). Biosurfactant Producing Microbes and their Potential Applications: A Review. Crit. Rev. Environ. Sci. Technol..

[137] Kang S.-W., Kim Y.-B., Shin J.-D., Kim E.-K. (2009). Enhanced Biodegradation of Hydrocarbons in Soil by Microbial Biosurfactant, Sophorolipid. Appl. Biochem. Biotechnol..

[138] Mulligan C.N., Yong R.N., Gibbs B.F. (2001). An evaluation of technologies for the heavy metal remediation of dredged sediments. J. Hazard. Mater..

[139] Reis R.S., Pacheco G.J., Pereira A.G., Freire D.M.G. (2013). Biodegradation—Life of Science. Biodegradation—Life of Science.

[140] Lang S., Wagner F., Kosaric N., Cairns W.L., Gray N.C.C. (1987). Structure and properties of biosurfactants. Biosurfactants and Biotechnology.

[141] Teichmann B., Linne U., Hewald S., Marahiel M.A., Bölker M. (2007). A biosynthetic gene cluster for a secreted cellobiose lipid with antifungal activity from Ustilago maydis. Mol. Microbiol..

[142] Cooper D.G., Paddock D.A. (1984). Production of a Biosurfactant from Torulopsis bombicola. Appl. Environ. Microbiol..

[143] Kitamoto D., Yanagishita H., Shinbo T., Nakane T., Kamisawa C., Nakahara T. (1993). Surface active properties and antimicro-bial activities of mannosylerythritol lipids as biosurfactants produced by Candida antarctica. J. Biotechnol..

[144] Suwansukho P., Rukachisirikul V., Kawai F. (2008). Production and applications of biosurfactant from Bacillus subtilis MUV4. Songklanakarin J. Sci. Technol..

[145] Begley M., Cotter P.D., Hill C., Ross R.P. (2009). Identification of a Novel Two-Peptide Lantibiotic, Lichenicidin, following Rational Genome Mining for LanM Proteins. Appl. Environ. Microbiol..

[146] Desai J.D., Banat I.M. (1997). Microbial production of surfactants and their commercial potential. Microbiol. Mol. Biol. Rev..

[147] Banat I.M., Franzetti A., Gandolfi I., Bestetti G., Martinotti M.G., Fracchia L., Smyth T.J., Marchant R. (2010). Microbial biosurfactants production, applications and future potential. Appl. Microbiol. Biotechnol..

[148] Lai C.-C., Huang Y.-C., Wei Y.-H., Chang J.-S. (2009). Biosurfactant-enhanced removal of total petroleum hydrocarbons from contaminated soil. J. Hazard. Mater..

[149] Zouboulis C.P.D., Piquero-Martín J. (2003). Update and Future of Systemic Acne Treatment. Dermatology.

[150] Aşçı Y., Nurbaş M., Açıkel Y.S. (2008). A comparative study for the sorption of Cd(II) by K-feldspar and sepiolite as soil components, and the recovery of Cd(II) using rhamnolipid biosurfactant. J. Environ. Manag..

[151] Ławniczak Ł., Marecik R., Chrzanowski Ł. (2013). Contributions of biosurfactants to natural or induced bioremediation. Appl. Microbiol. Biotechnol..

[152] Singh A., Van Hamme J.D., Ward O.P. (2007). Surfactants in microbiology and biotechnology: Part 2. Application aspects. Biotechnol. Adv..

[153] Satpute S.K., Banpurkar A.G., Dhakephalkar P.K., Banat I.M., Chopade B.A. (2010). Methods for investigating biosurfactants and bioemulsifiers: A review. Crit. Rev. Biotechnol..

[154] Sekhon-Randhawa K.K., Kosaric N., Sukan F.V. (2014). Biosurfactants produced by genetically manipulated microorganisms: Challenges and opportunities. Biosurfactants.

[155] Uzoigwe C., Burgess J.G., Ennis C.J., Rahman P.K.S.M. (2015). Bioemulsifiers are not biosurfactants and require different screening approaches. Front. Microbiol..

[156] Maneerat S. (2005). Biosurfactants from marine microorganisms. Songklanakarin J. Sci. Technol..

[157] Gutierrez T., Mulloy B., Bavington C., Black K., Green D.H. (2007). Partial purification and chemical characterization of a glycoprotein (putative hydrocolloid) emulsifier produced by a marine bacterium Antarctobacter. Appl. Microbiol. Biotechnol..

[158] Gutierrez T., Mulloy B., Black K., Green D.H. (2007). Glycoprotein emulsifiers from two marine Halomonas species: Chemical and physical characterization. J. Appl. Microbiol..

[159] Schulz D., Passeri A., Schmidt M., Lang S., Wagner F., Wray V., Gunkel W. (1991). Marine biosurfactants, I. Screening for bio-surfactants among crude oil degrading marine microorganisms from the North Sea. Zeitschrift für Naturforschung C.

